# High power tunable Raman fiber laser at 1.2 μm waveband

**DOI:** 10.1007/s12200-024-00105-7

**Published:** 2024-01-15

**Authors:** Yang Zhang, Jiangming Xu, Junrui Liang, Jun Ye, Sicheng Li, Xiaoya Ma, Zhiyong Pan, Jinyong Leng, Pu Zhou

**Affiliations:** 1 College of Advanced Interdisciplinary Studies, National University of Defense Technology, Changsha, 410073 China; 2 Nanhu Laser Laboratory, National University of Defense Technology, Changsha, 410073 China; 3 Hunan Provincial Key Laboratory of High Energy Laser Technology, National University of Defense Technology, Changsha, 410073 China

**Keywords:** Phosphosilicate fiber, Raman fiber laser, 1.2 μm waveband, Wavelength tunable

## Abstract

Development of a high power fiber laser at special waveband, which is difficult to achieve by conventional rare-earth-doped fibers, is a significant challenge. One of the most common methods for achieving lasing at special wavelength is Raman conversion. Phosphorus-doped fiber (PDF), due to the phosphorus-related large frequency shift Raman peak at 40 THz, is a great choice for large frequency shift Raman conversion. Here, by adopting 150 m large mode area triple-clad PDF as Raman gain medium, and a novel wavelength-selective feedback mechanism to suppress the silica-related Raman emission, we build a high power cladding-pumped Raman fiber laser at 1.2 μm waveband. A Raman signal with power up to 735.8 W at 1252.7 nm is obtained. To the best of our knowledge, this is the highest output power ever reported for fiber lasers at 1.2 μm waveband. Moreover, by tuning the wavelength of the pump source, a tunable Raman output of more than 450 W over a wavelength range of 1240.6–1252.7 nm is demonstrated. This work proves PDF’s advantage in high power large frequency shift Raman conversion with a cladding pump scheme, thus providing a good solution for a high power laser source at special waveband.

**Graphical Abstract:**

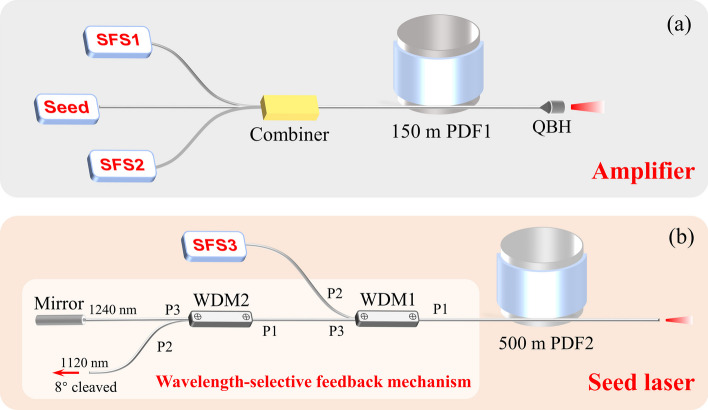

**Supplementary Information:**

The online version contains supplementary material available at 10.1007/s12200-024-00105-7.

## Introduction

Laser sources operating at 1.2 μm waveband have some unique applications in photodynamic therapy, biomedical diagnosis and oxygen sensing [1–5]. Additionally, they can be adopted as pump sources for mid-infrared optical parametric generation as well as visible light generation by frequency doubling [6–9]. Laser generation at 1.2 μm waveband has been achieved with different solid-state lasers including semiconductor laser [10, 11], diamond Raman laser [12, 13], and fiber laser [14, 15]. Among these three types, the semiconductor laser has the most compact structure, but its output power at this waveband is limited to watt-level [11]. The diamond Raman laser has achieved kilowatt-level output at 1.2 μm waveband, but the laser system is complicated [16]. By contrast, fiber laser, thanks to its simple structure and high brightness, is a great choice for 1.2 μm waveband laser generation. Currently, fiber lasers operating at 1.2 μm waveband can be divided into two groups in terms of the lasing mechanism. The first one adopts some special ion-doped fibers such as bismuth-doped fiber or holmium-doped fluoride fiber to provide optical gain at this waveband directly [17–19]. However, due to the relatively small gain coefficient and difficulties in the fiber fabrication process, the output power of these lasers at 1.2 μm waveband is limited within ten-watt level [18, 19]. The other one utilizes stimulated Raman scattering effect in passive fiber to convert the beam from a high power ytterbium-doped fiber laser (YDFL) into 1.2 μm waveband [20–23]. For common passive fiber like pure silica fiber or germanium-doped fiber, the Raman shift is about 13.2 THz, and three-order cascaded Raman conversion is needed for 1.2 μm laser generation. The phosphorus-doped fiber (PDF), however, has a strong phosphorus-related Raman peak at frequency shift of about 40 THz [24, 25]. Only one order of Raman conversion is needed to convert YDFL outputs into 1.2 μm waveband [26, 27]. The reduction of Raman orders not only simplifies the structure but also increase the conversion efficiency, making PDF a better choice for large frequency shift wavelength conversion.

In 1997, Dianov et al. converted 1064 nm pump light into 1240 nm, through one order Raman conversion in a PDF-based Raman fiber laser (RFL) [28]. Since then, PDF has been widely adopted in RFLs for large frequency shift wavelength conversion [29–35]. In 2003, Xiong et al. demonstrated 11.35 W output power at 1248 nm in a 300 m PDF-based RFL [36]. In 2019, by reducing the length of PDF to 30 m and adopting high power YDFL as a pump source, Dong et al. boosted the Raman output power at 1.24 μm to 206.7 W [37]. Further power scaling is limited by the fast-growing parasitic lasing at 1.12 μm (generated by the silica-related Raman peak). It should be noted that the reported 1.2 μm RFLs above are all core-pumped with small core diameter PDF (core diameter about 5 μm), and the further power scaling is inherently limited by the relatively low power capacity and low threshold power of high order Raman Stokes light. Besides, as the phosphorus-related Raman peak has relatively lower gain coefficient and narrower gain bandwidth than those of the silica-related Raman peak, high reflective fiber Bragg grating at specific wavelength which exactly matches the frequency shift of the phosphorus-related Raman peak is employed to suppress the silica-related Raman emission [38]. Thus, it’s difficult for PDF-based RFL to achieve tunable output at 1.2 μm waveband.

In this paper, we present a PDF-based high power wavelength tunable RFL at 1.2 μm waveband. At first, by using a low power tunable superfluorescent fiber source (SFS) as pump and employing a wavelength-selective feedback mechanism to suppress the silica-related Raman emission, we obtain about 23 W Raman output over a tuning range of 1240.6–1252.7 nm in a PDF-based random RFL. Then, by adopting this tunable random RFL as seed laser, with two high power tunable SFSs as pump sources, and 150 m specially designed large mode area PDF as gain medium, we build a high power cladding-pumped RFL. More than 450 W Raman output over the tuning range of 1240.6–1252.7 nm is demonstrated. When the RFL operates at 1252.7 nm, the maximum output signal power is up to 736 W. To the best of our knowledge, this is the highest output power ever reported for fiber lasers at 1.2 μm waveband.

## Experimental principle and setup

**Fig. 1:**
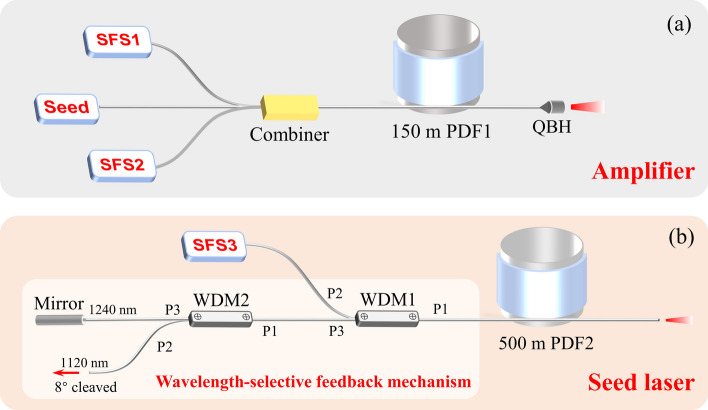
Experimental setup of the **a** high power tunable Raman fiber amplifier and **b** tunable random Raman fiber seed laser at 1.2 μm waveband. PDF, phosphorus-doped fiber; QBH, quartz block head; WDM, wavelength division multiplexer; SFS, superfluorescent fiber source; P1, port 1; P2, port 2; P3, port 3

The experimental setup of the proposed high power tunable RFL at 1.2 μm waveband is shown in Fig. 1a. It consists of a tunable seed laser at 1.2 μm waveband, two high power pump sources, a (2 + 1) × 1 pump/signal combiner, 150 m PDF (PDF1), and a quartz block head. The seed laser is a tunable random RFL at 1.2 μm waveband, the detailed structure of which will be illustrated in the next paragraph. The pump sources are two high power tunable SFSs, which can totally deliver up to 1.4 kW power over a tuning range of 1065–1085 nm [39]. The output fibers of the two pump sources have the same core size of 20 μm. The Raman gain fiber is 150 m homemade triple-clad PDF (PDF1), whose core and inner cladding diameters are 29 and 56 μm, respectively. The numerical apertures of the core and inner-cladding area are 0.08 and 0.17, respectively. Only the core is doped with phosphorus, to induce a strong phosphorus-related Raman peak at a frequency shift of about 40 THz. The transmission loss of the core area is about 1.1 dB/km at 1.24 μm. The seed laser is coupled into the core and the pump sources are coupled into the cladding of the PDF1 through a (2 + 1) × 1 pump/signal combiner. In the combiner, the core diameter of the signal fiber is 10 μm and that of the pump input fibers is 20 μm. The output fiber is the PDF1. A home-made quartz block head is employed to expand the high power light beam and suppress Fresnel reflection.

**Fig. 2:**
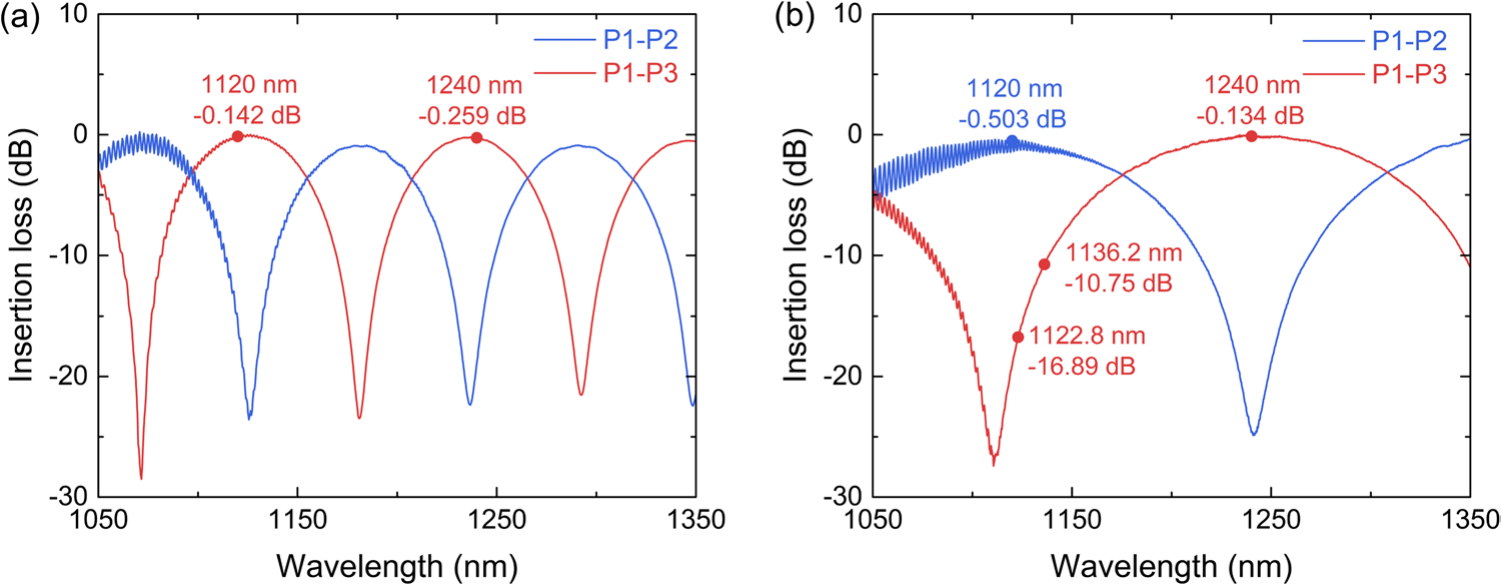
Transmission spectra of **a** WDM1 and **b** WDM2

The seed laser is a half-open-cavity random RFL, the schematic diagram of which is shown in Fig. 1b. The pump source is a low power tunable SFS (SFS3), which can deliver 50 W output power over a tuning range of 1065–1085 nm. The Raman gain fiber is 500 m PDF (PDF2), with a core diameter of 5 μm and a numerical aperture of 0.18. Between the SFS3 and the PDF2, a wavelength-selective feedback mechanism is employed to provide point feedback for the Raman signal at around 1.24 μm. The wavelength-selective feedback mechanism consists of two wavelength division multiplexers (WDM1 and WDM2) and a broadband mirror. The transmission spectra of these two WDMs are displayed in Fig. 2. With this wavelength-selective feedback mechanism, backward scattered silica-related Raman light and phosphorus-related Raman light are both guided into the WDM2 through the P3 port of WDM1, then silica-related Raman light is exported through the P2 port of WDM2 while the phosphorus-related Raman light passes through the P3 port of WDM2 and is reflected back by the broadband mirror. As a result, phosphorus-related Raman emission at around 1.24 μm is stimulated while the silica-related Raman emission at around 1.12 μm is suppressed. Moreover, the pigtail fibers of the two WDMs and broadband mirror have the same core diameters of 10 μm. Due to the mismatch of the mode field diameter, there is a small splicing loss (about 0.43 dB) between the WDM1 and the PDF2.

## Result and discussion

### Characteristics of the tunable seed laser

**Fig. 3:**
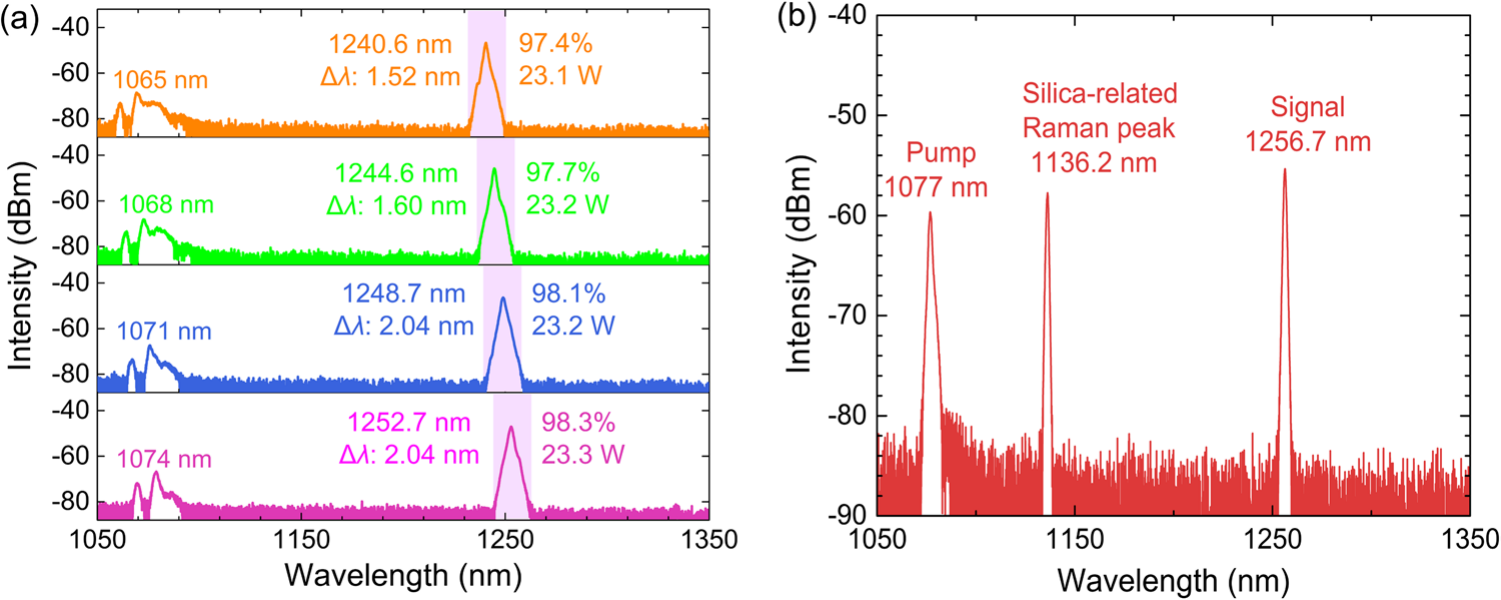
Output spectra of the tunable random RFL under pump wavelengths of **a** 1065–1074 nm and **b** 1077 nm (Δ*λ* refers to the 3 dB linewidth)

By changing the central wavelength of the SFS, the output wavelength of the random RFL seed source can be flexibly tuned. When the pump wavelength is increased from 1065 to 1074 nm with a fixed gap of 3 nm, the output signal can be tuned from 1240.6 to 1252.7 nm. The corresponding output spectra at maximum signal power are presented in Fig. 3a. A signal of more than 23 W, with spectral purity of > 97%, is demonstrated over the tuning range of 1240.6–1252.7 nm. The 3 dB linewidth of the tunable seed signal ranges from 1.52 to 2.04 nm. When the pump wavelength is further increased to 1077 nm, as shown in Fig. 3b, a strong silica-related Raman peak at 1136.2 nm is generated, thus restricting the further wavelength extension of the tunable random RFL. The generation of the silica-related Raman peak is related to the limited bandwidth of the wavelength-selective feedback mechanism. For the initial pump wavelength of 1065 nm, the silica-related Raman peak is at around 1122.8 nm. As shown in Fig. 2b, the insertion loss between P1 and P3 of the WDM2 at 1122.8 nm is 16.89 dB. The backward scattered light component at 1122.8 nm is mostly exported from the P2 port of WDM2; the feedback at this wavelength is almost negligible. As pump wavelength increases, the wavelength of the silica-related Raman emission increases correspondingly, and the transmittance also increases. When the pump wavelength is increased to 1077 nm, the insertion loss of the corresponding silica-related Raman peak at 1136.2 nm decreases to 10.75 dB. The feedback at 1136.2 nm is non-negligible. As a result, a strong silica-related Raman peak is generated. To better explain the gain competition between the silica-related Raman emission and the phosphorus-related Raman emission in this half-open cavity random RFL, a simple simulation is carried out (See Supplement 1 for more details).

**Fig. 4:**
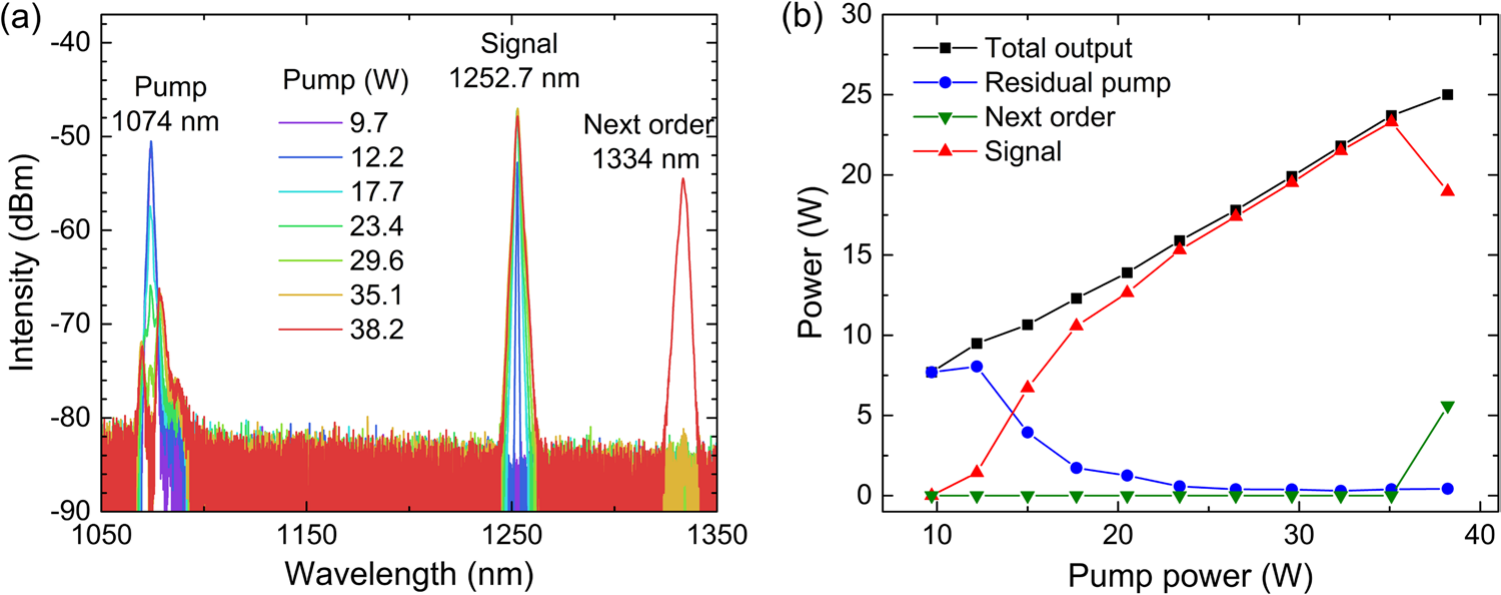
**a** Spectral and **b** power evolution characteristics of the tunable random RFL under pump wavelength of 1074 nm

Figure 4a and b, respectively, display the detailed spectral and power evolution characteristics of the tunable random RFL under pump wavelength of 1074 nm. When the pump power reaches 12.2 W, a phosphorus-related Raman peak at 1252.7 nm is generated. As the pump power continues to increase, more of the pump output is converted into the 1252.7 nm signal. When the pump power increases to 35.1 W, the signal power reaches a maximum of 23.3 W, and only 0.4 W pump power remains unconverted. Thanks to the good temporal stability of the SFS, the pump power is sufficiently converted and the spectral purity of the signal is up to 98.3% [40–43]. Further increase of signal power is restricted by the fast growth of the next order silica-related Raman emission at 1334 nm.

### Characteristics of the high power tunable Raman fiber amplifier

**Fig. 5:**
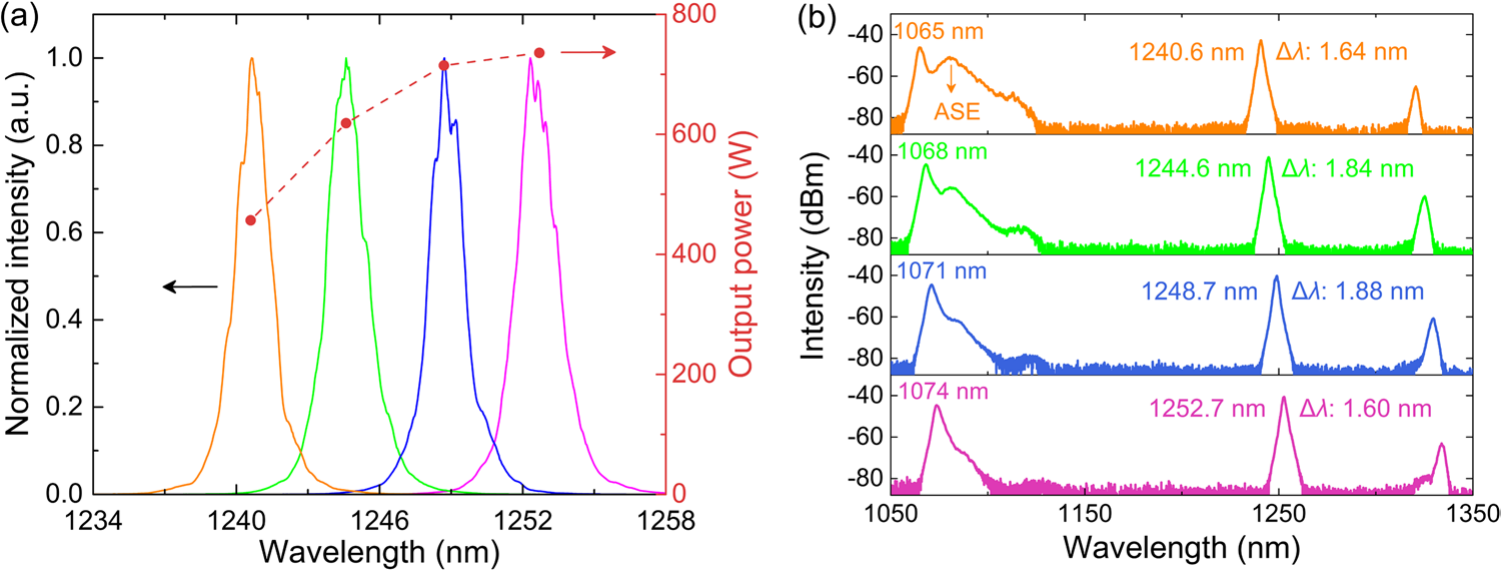
**a** Maximum output power and normalized output spectra at different signal wavelengths. **b** Full output spectra at different signal wavelengths in dB scale (Δ*λ* refers to the 3 dB linewidth)

By synchronously tuning the pump wavelength in the amplifier from 1065 to 1074 nm, the tunable signals in the range of 1240.6 to 1252.7 nm are boosted to > 450 W. The maximum output power and corresponding normalized output spectra at different signal wavelengths are presented in Fig. 5a. The full output spectra in logscale are presented in Fig. 5b. The 3 dB linewidth of the amplifier Raman signal varies from 1.60 to 1.88 nm. When the pump wavelength is 1074 nm, the 1252.7 nm Raman signal is amplified to 735.8 W. As the pump wavelength decreases, the output signal power drops significantly. When the pump wavelength is 1065 nm, the output power of the 1240.6 nm signal is 456.7 W. The relatively low output signal power under short pump wavelength is related to the 1080 nm amplified spontaneous emission (ASE) spectral component. As shown in Fig. 5b, when the high power SFS pump source is tuned to 1065 nm, a broad band ASE envelope around 1080 nm is observed. Amplified by the boson peak in the PDF, part of the 1065 nm pump output is converted into the 1080 nm ASE spectral component [44, 45]. As the pump wavelength increases, the intensity of the 1080 nm ASE spectral component decreases significantly, less pump light is converted into the 1080 nm ASE spectral component. Consequently, the output signal power at 1.2 μm is higher than that under longer pump wavelength.

**Fig. 6:**
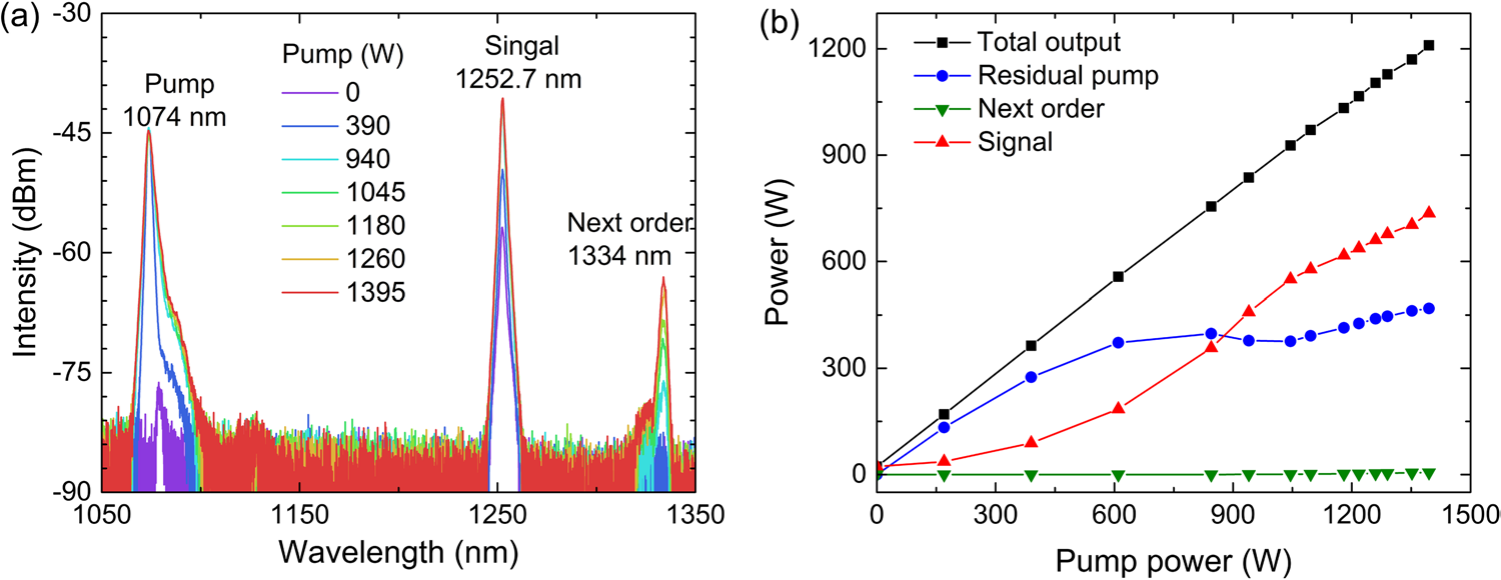
**a** Spectral and **b** power evolution characteristics of the high power tunable Raman fiber amplifier at pump wavelength of 1074 nm

Figure 6a and b present the spectral and power evolution characteristics, respectively, of the high power Raman amplifier under pump wavelength of 1074 nm. The initial input signal power at 1252.7 nm seed source output is 23.3 W. Under low pump power, most of the pump output passes through the fiber directly without being unconverted. As the pump power increases, more of it is converted into the 1252.7 nm signal. When the pump power reaches 940 W, the signal power exceeds the residual pump power. Meanwhile, a new spectral peak at 1334 nm is observed, which is the next order Stokes light of the 1252.7 nm signal related to the silica-related Raman peak. When the pump power reaches its maximum of 1395 W, the output power of the 1252.7 nm signal increases to 735.8 W, the next order Raman emission at 1334 nm reaches 5.5 W, and the residual pump power is 468.6 W. The optical-to-optical conversion efficiency at maximum pump power is 76.9% and 51.1% of the signal power for absorbed pump power and for launched pump power, respectively. The relatively low conversion efficiency is mainly due to the high quantum defect, low Raman gain coefficient, and large clad-to-core ratio. Compared to values relating to the common RFL based on 13.2 THz Raman peak, the quantum defect of the 1.2 μm waveband RFL is three times higher and the gain coefficient of the 40 THz Raman peak is lower. The output signal power as well as the conversion efficiency could be further increased through the optimization of fiber parameters, such as by decreasing the clad-to-core diameter ratio and increasing the concentration of phosphorus dopant. The next order Raman laser could be suppressed by inserting chirped and tilted fiber Bragg gratings between the seed laser and the Raman amplifier [46].

## Conclusion

In conclusion, we report a PDF-based cladding-pumped high power tunable RFL at 1.2 μm waveband. Seeded by a home-made tunable random RFL and pumped by two high power tunable SFSs, up to 735.8 W Raman signal at wavelength 1252.7 nm is obtained under the maximum pump power of 1395 W. To the best of our knowledge, this is the highest output power ever reported for fiber lasers at 1.2 μm wave. Moreover, by adjusting the wavelength of the pump source, we demonstrate a tunable Raman output of power over 450 W over a wavelength range of 1240.6–1252.7 nm. The output signal power and the conversion efficiency are supposed to be furtherly improved through the optimization of fiber parameters, such as reducing clad-to-core diameter ratio and increasing the concentration of phosphorus dopant. This work is of significance in extending the spectral range of high power fiber lasers.

### Supplementary Information

Below is the link to the electronic supplementary material.

Supplementary file1 (PDF 254 KB)

## Data Availability

Data underlying the results presented in this paper are not publicly available at this time but may be obtained from the authors upon reasonable request.
