# Supplementary material for: High power tunable Raman fiber laser at 1.2 μm waveband
Source: Front Optoelectron. 2024 Jan 15;17(1):1. doi: 10.1007/s12200-024-00105-7 (PMC10789707; doi:10.1007/s12200-024-00105-7)
Supplement: Supplementary file 1 — Supplementary file1 (PDF 254 KB) [file 12200_2024_105_MOESM1_ESM.pdf]

Supporting Information

High power tunable Raman fiber laser at 1.2 μm wavelength

Yang Zhang[1], Jiangming Xu[1,*], Junrui Liang[1], Jun Ye[1,2,3], Sicheng Li[1], Xiaoya Ma[1], Zhiyong Pan[1,2,3], Jinyong Leng[1,2,3], Pu Zhou[1,*]

[1]College of Advanced Interdisciplinary Studies, National University of Defense Technology, Changsha 410073, China.

[2]Nanhu Laser Laboratory, National University of Defense Technology, Changsha 410073, China.

[3]Hunan Provincial Key Laboratory of High Energy Laser Technology, National University of Defense Technology, Changsha 410073, China.

*E-mail: jmxu1988@163.com; zhoupu203@163.com

Figure S1 displays the Raman gain spectra of the phosphorus-doped silica fiber (PDF2). The gain coefficient of the silica-related Raman peak is higher than the phosphorus-related Raman peak. To suppress the silica-related Raman emission, in the tunable random Raman fiber laser (RRFL), we adopted a wavelength sensitive feedback mechanism to provide feedback for the phosphorus-related Raman emission. Restricted by the working bandwidth of the wavelength sensitive mechanism, the silica-related Raman emission can only be suppressed within certain wavelength range. Consequently, the wavelength tuning range of the tunable random Raman fiber seed laser is limited.

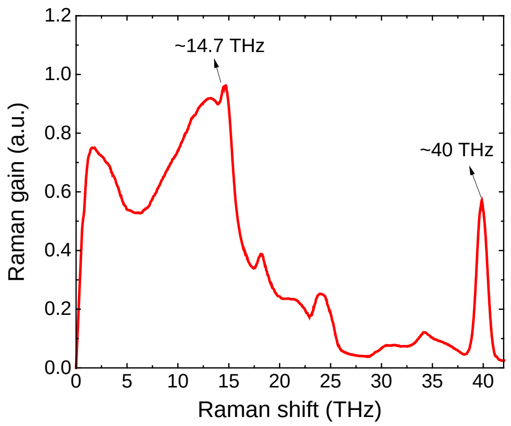

Fig. S1. Raman gain spectrum of the phosphorus-doped fiber (PDF2) [1].

To better explain the gain competition between silica-related Raman peak and phosphorus-related Raman peak, we calculated the power evolution characteristics of the silica-related Raman emission and phosphorus-related Raman emission in the half-open-cavity RRFL. The calculations are based on the general power balanced model [2,3]:

$$\pm\frac{\mathrm{d}P_0^\pm}{\mathrm{d}z}=-\alpha_0P_0^\pm-g_{R1}\frac{\upsilon_0}{\upsilon_1}P_0^\pm(P_1^++P_1^-+\Gamma_1)+\varepsilon_0P_0^{\mathrm{m}},\tag{1}$$

$$\pm\frac{\mathrm{d}P_1^\pm}{\mathrm{d}z}=-\alpha_1P_1^\pm+g_{R1}(P_1^\pm+0.5\Gamma_1)(P_0^++P_0^-)-g_{R2}\frac{\upsilon_1}{\upsilon_2}P_1^\pm(P_2^++P_2^-+\Gamma_2)+\varepsilon_1P_1^{\mathrm{m}},\tag{2}$$

$$\pm\frac{\mathrm{d}P_2^\pm}{\mathrm{d}z}=-\alpha_2P_2^\pm+g_{R2}(P_2^\pm+0.5\Gamma_2)(P_1^++P_1^-)+\varepsilon_2P_2^{\mathrm{m}},\tag{3}$$

$$\Gamma_j=4h\upsilon_j\Delta\upsilon_j\left\{1+\frac{1}{\exp\left[h(\upsilon_{j-1}-\upsilon_j)/k_BT\right]-1}\right\},\ \ j=1,2,\tag{4}$$

where the lower indices 0, 1, and 2 refer to the corresponding terms to pump, signal light and the next order Raman Stokes light, while upper indices + and – refer to forward and backward propagating waves, respectively. P is the power, α is the attenuation coefficient, v is the frequency, Δv is the Raman amplification bandwidth, ε is the backscattering coefficient, derived as α multiplied by backscattering factor Q (approximately to 0.0017 for the phosphosilicate fiber). T (=298K) is the absolute temperature, k_B is the Boltzmann's constant, h is the Plank's constant. g_R is the effective Raman gain coefficient. And the boundary conditions are as follow:

$$P_0=P_{in},\tag{5}$$

$$P_j^+(0)=P_j^-(0)R_{Lj},\ \ j=1,2,\tag{6}$$

$$P_j^-(L)=P_j^+(L)R_{Rj},\ \ j=1,2.\tag{7}$$

where R_{L1} is the reflectivity of the mirror and R_{R1} the reflectivity of fiber ends.

Table S1 Fiber parameters in power evolution characteristics calculation

Phosphorus-related Raman emission		silica-related Raman emission	
Symbol	Value	Symbol	Value
λ_0, λ_1, λ_2,	1077, 1256.7, 1338.5 nm	λ_0, λ_1, λ_2,	1077, 1136.2, 1202.7 nm
α_0, α_1, α_2,	1.56, 1.03, 0.87 dB/km	α_0, α_1, α_2,	1.56, 1.03, 0.87 dB/km
R_{L1}, R_{R1}	0.75, 2×10^{-5}	R_{L1}, R_{R1}	0.0075, 2×10^{-5}
g_{R1}, g_{R2}	0.7, 1.2 $km^{-1}\cdot W^{-1}$	g_{R1}, g_{R2}	1.2, 1.2 $km^{-1}\cdot W^{-1}$
L	500 m	L	500 m

Based on the proposed power balanced model above, we firstly calculate the power evolution characteristics of phosphorus-related Raman emission as well as silica-related Raman emission in this half-open cavity RRFL. Simulation parameters in this calculation are summarized in Table 1. Considering the insertion loss of the wavelength division multiplexer and the fiber splicing loss induced by mode area mismatch, R_{L1} is set to 0.75 for the phosphorus-related Raman emission at 1256.7 nm while for the silica-related Raman emission at 1136.2 nm it's set to 0.0075. The calculated power evolution characteristics of phosphorus-related Raman emission and silica-related Raman emission are shown in Fig. S2(a) and S2(b) respectively. For the convenience of comparison, we define the pump power at which the residual pump power begins to drop as the threshold power of signal. As shown in the figure, the threshold powers of the phosphorus-related Raman emission and silica-related Raman emission are both about 18 W. Although the simulation results don't perfectly agree with the experimental results in quantity, it could help explain the generation of silica-related Raman peak at 1136.2 nm in the tunable RRFL under pump wavelength of 1077 nm.

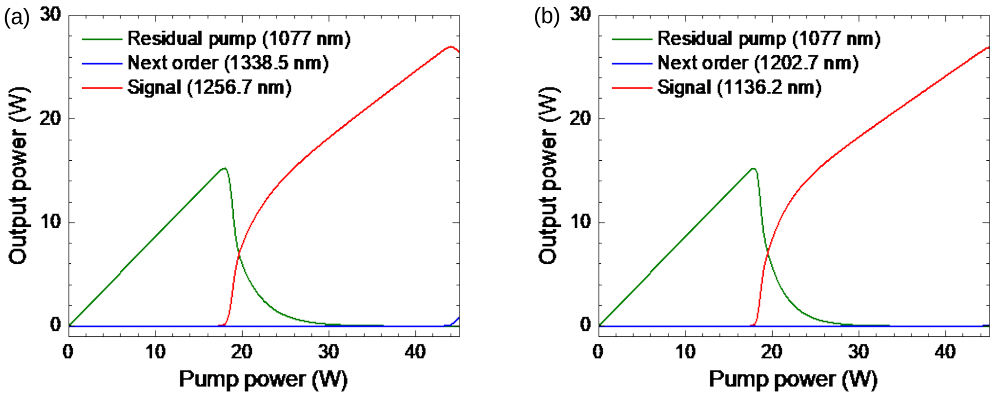

Fig. S2. The power evolution characteristics of (a) phosphorus-related Raman emission and (b) silica-related Raman emission in this half-open-cavity RRFL at pump wavelength of 1077 nm.

Furtherly, we calculate the threshold power of phosphorus-related Raman emission and silica-related Raman emission in this tunable RRFL under different pump wavelengths. The reflectivity of the wavelength-sensitive mechanism at different wavelength can be obtained from the transmission spectra of the two wavelength division multiplexers. The calculated results are presented in Fig. S3. At short pump wavelength, the threshold power of phosphorus-related Raman emission is lower than the silica-related Raman emission. As the pump wavelength increases, the reflectivity R_{L1} for the silica-related Raman emission increases significantly, and for the phosphorus-related Raman emission, R_{L1} slightly decreases. As a result, the threshold power of phosphorus-related Raman emission is higher than the silica-related Raman emission when the pump wavelength is longer than 1077 nm. This simulation results could help explain the wavelength tuning restriction imposed by silica-related Raman emission in our tunable seed laser at 1.2 μm wavelength band.

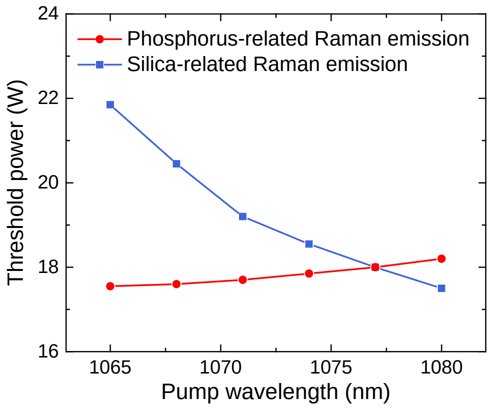

Fig. S3. The threshold power of phosphorus-related Raman emission and silica-related Raman emission in this half-open cavity RRFL under different pump wavelengths.

Reference

1. J. Ye, Y. Zhang, J. Xu, J. Song, T. Yao, H. Xiao, J. Leng, and P. Zhou, "Investigations on the extreme frequency shift of phosphosilicate random fiber laser," J. Lightwave Technol. 38(14), 3737-3744 (2020).
2. D. V. Churkin, A. E. El-Taher, I. D. Vatnik, J. D. Ania-Castañón, P. Harper, E. V. Podivilov, S. A. Babin, and S. K. Turitsyn, "Experimental and theoretical study of longitudinal power distribution in a random DFB fiber laser," Opt. Express 20(10), 11178-11188 (2012).
3. J. Ye, J. Xu, J. Song, Y. Zhang, H. Zhang, H. Xiao, J. Leng, and P. Zhou, "Pump scheme optimization of an incoherently pumped high-power random fiber laser," Photon. Res. 7(9), 977-983 (2019).
